# An Introduction and Brief Overview of Psychoanalysis

**DOI:** 10.7759/cureus.45171

**Published:** 2023-09-13

**Authors:** Martin Tarzian, Mariana Ndrio, Adegbenro O Fakoya

**Affiliations:** 1 Psychiatry, University of Medicine and Health Sciences, Basseterre, KNA; 2 Psychiatry and Behavioral Sciences, University of Medicine and Health Sciences, Basseterre, KNA; 3 Cellular Biology and Anatomy, Louisiana State University Health Sciences Center, Shreveport, USA

**Keywords:** karen horney, erik erikson, alfred adler, carl jung, sigmund freud, psychology, unconscious, psychoanalysis

## Abstract

The field of psychology has been shaped by the views and perspectives of Sigmund Freud and his former students: Carl Jung, Alfred Adler, Erik Erikson, and Karen Horney. These psychologists and their respective schools of thought provide distinct views on psychology and influences on personality and psychosocial development. The foundation for psychoanalysis was laid by Freud, his work on the unconscious mind, and his emphasis on early childhood experiences. His students also made substantial contributions that expanded, sharpened, and challenged his theories. This review will begin by highlighting the work of Sigmund Freud; it will then compare his theories with the theories of his students. This review will introduce and bring attention to the most important figures of psychoanalysis and give a brief overview of their theories.

## Introduction and background

Sigmund Freud (Born May 6th, 1856) was an Austrian neurologist widely known as the father of psychoanalysis. His revolutionary theories, thoughts, and challenges to the field of psychology have changed how theorists understand the mind and how psychologists treat their patients [1]. Throughout his life, he worked fervently and tirelessly to advance his theories, beginning in the late 19th century and continuing until he died in 1939. Today, most clinical psychologists and psychiatrists roll their eyes when Freud’s ideas are brought up. However, his impact on shaping the theoretical and practical approaches to the human mind and reasoning for behavior cannot be understated or forgotten [2].

Freud attended medical school at the University of Vienna and graduated as a neurologist [1,2]. He was interested in the brain’s anatomy and how the nervous system works in relation to the human body. Time and time again, his research led him to query and ponder the relationship between the human mind and the physical ailments his patients would experience [3]. He began to question whether the symptomology he witnessed in his patients came from the illness within the brain or the mind. As time passed, Freud developed a passion for understanding the complexities of the mind and psyche, eventually leading him to pursue his pioneering work of *psychoanalysis *[3].

As his passion and interest in the human mind increased, Sigmund Freud shifted from treating neurological diseases to treating individuals suffering from psychological issues such as depression and anxiety [3]. This switch gradually occurred in the late 1800s. By the 1900s, Freud was solely focused on using psychoanalysis to explain anomalies of human nature [3,4]. While working at the University of Vienna, his professor, Dr. Josef Breuer, introduced Sigmund Freud to a fascinating case. The patient, referred to as “Anna O,” experienced a range of physical symptoms such as visual disturbances, hallucinations, partial paralysis, and speech problems with no apparent physical cause [4]. Dr. Breuer found that her symptoms improved when he helped her recover memories of traumatic experiences that she had repressed from conscious awareness, which he called the “Cathartic Method” [4]. Unsurprisingly, Sigmund Freud, who was already fascinated with anomalies of the human mind, feverishly began exploring the possible psychological phenomena that could explain Anna Os’s symptomology. Later, in 1895, Freud and Breuer published a book discussing Anna’s case and other similar clinical cases called “Studies in Hysteria.” This case is significant because it laid the foundation for developing Freud’s influential ideas about psychoanalysis [5,6].

Continuing his work with Breuer, Freud finally derived his groundbreaking theory about “The Unconscious,” proposing that repressed memories and desires influence behavioral and emotional states. A revolutionary concept at the time, this theory would permanently leave its imprint on psychological thinking for centuries to come [6,7]. This time can also be considered the period when Freud abandoned neurology as a field and focused solely on practicing psychoanalysis. While initially he used the method of hypnosis taught to him by Charcot, Freud recognized that he needed a better way of addressing his patients’ unconscious desires and memories. Thus, he developed the “Free Association,” in which patients were encouraged to speak freely about their thoughts and feelings without censorship or self-editing [7,8]. Through this technique, Freud believed that he uncovered the unconscious thoughts and memories that were the root cause of many psychological phenomena and anomalies [7,8].

In 1899, Sigmund Freud released his groundbreaking book “The Interpretation of Dreams.” He proposed a new theory on the meaning behind dreams and how they relate to the unconscious [9-11]. In his book, he argued that dreams portal into humankind’s hidden desires and emotions and provide insight into any underlying conflicts within the unconscious mind. At the start of the 1900s, Freud had established himself as the master of addressing the unconscious [1-3]. As time passed, his theories continued to grow more rambunctious and ambitious in their claims of explaining human behavior. His work revolutionized how we view the human psyche and opened many avenues for further exploration [9].

In the early 1900s, Sigmund Freud had established himself at the frontier of psychological thinking and began writing about the Oedipus complex [1-3]. This theory claims that young children have an unconscious sexual desire toward the parent of the opposite sex. The Oedipal complex has come to be known as Sigmund Freud’s most controversial theory and is often joked about when referenced in popular media [12]. He also formulated the tripartite theory involving the id, ego, and superego - a theory still taught today to explain different facets of the human psyche. Over the next decade, Freud dedicated himself to improving these theories [12]. In 1902, Sigmund Freud formed the Vienna Psychoanalytic Society, and it quickly became a popular hub for the exploration and development of his theories. He also began instructing other mental health professionals in psychoanalysis, leading to its increased spread across Europe and the United States in the following years [1,2]. His work at the Vienna Psychoanalytic Society, along with his time spent teaching, are regarded as the reasons that psychoanalysis quickly globalized at the turn of the 20th century.

Despite the initial success of psychoanalysis, Freud’s theories were controversial and faced criticism from many quarters. Some critics accused Freud of being overly focused on sex and of exaggerating the influence of the unconscious mind. Others argued that psychoanalysis lacked scientific validity and was based on anecdotal evidence rather than empirical data [13]. Ultimately, even his students challenged him and began their own schools of thought, which we will discuss later [13]. Nevertheless, Freud’s influence on psychology and culture cannot be overstated. His theories continue to be the subject of debate and research, and psychoanalysis remains a popular and influential field of study. Freud’s discovery of psychoanalysis paved the way for a new understanding of the human mind and continues to shape our understanding of psychology and society [1-3].

Freud’s legacy extends beyond his contributions. His circle of inspired and loyal pupils continued to push and advance the field. Together, they have expanded the principles of psychoanalysis. These students, united in their respect and admiration for Freud, carry his ideas forward, establishing psychoanalysis as a legitimate and influential approach to understanding the human mind. By continuing to explore and apply psychoanalytic principles, we can gain a deeper understanding of ourselves, our actions, and the intricate workings of the human mind. This review aims to emphasize the contributions of psychoanalysis, from Sigmund Freud to his students Carl Jung, Alfred Adler, Erik Erikson, and Karen Horney.

## Review

Freud’s Free Association Technique

Free association is the fundamental technique of addressing the unconscious in psychoanalysis. The method allows patients to freely express their thoughts, feelings, and emotions without censoring themselves. Commonly, the patient lies on a couch in the physician’s office and is made to feel very comfortable. This lowers the patient’s guard and thus the ego’s guard, allowing the mind to truly express its reservations [14]. The goal is to elicit the unconscious thoughts and memories contributing to psychological distress [14]. The theory behind free association is that unconscious thoughts and emotions may be painful, embarrassing, or socially unacceptable and thus remain submerged in the unconscious [14]. The patient is lulled into a state of comfort through free association, allowing these subconscious notions to come to light [14].

By bringing these unconscious thoughts and feelings into conscious awareness, the patient is allowed to address them and understand why they may be experiencing distress from them. The therapist can encourage the patient to talk about dreams, childhood memories, or anything that may bring the patient’s underlying issues to conscious awareness [15]. The therapist must listen without interrupting or imposing their own ideas, thus creating a safe space to express themselves openly and speak honestly [15].

Free association is not without its limitations and criticism. One of the criticisms of free association is that it relies too much on the therapist’s ability to interpret the patient’s unconscious thoughts and feelings. This is subjective, and different therapists may interpret the exact words or phrases differently [16]. Take, for instance, a dream depicting a tiger pursuing a gazelle in the depths of the jungle. One therapist might interpret the tiger as a symbol of the patient’s personal strength and confidence, while another therapist could perceive the gazelle as a representation of the patient’s apprehension regarding vulnerability to external forces. Determining which interpretation is objectively accurate is inherently elusive if not impossible. Another challenge with free association is that it is time-consuming and arduous. Patients may struggle to access their unconscious thoughts, feelings, and/or emotions, and it may take several months or even years to express themselves honestly and freely [16]. Despite these limitations, free association remains a fascinating approach to both therapy and addressing the unconscious. While it has limitations, free association remains an interesting tool in treating psychological disorders. It is a pivotal technique used by Sigmund Freud and other followers of psychoanalysis [15].

The Oedipal complex

First proposed by Sigmund Freud, the Oedipal complex is an essential concept within psychoanalysis. According to Freud, the Oedipal complex is a psychological phenomenon occurring between three and six in otherwise healthy children [17]. This desire is theorized to originate from the child’s need to satisfy sexual instincts, which are repressed by social norms and familial expectations [18-20]. It represents a child’s unconscious desire to own the same-sex parent and eliminate the other, whom they view as competition. The child develops hostility, jealousy, and anger toward the same-sex parent while experiencing love and sexual attraction toward the parent of the opposite sex [18-20].

The Oedipal complex has been criticized and debated outside and within the psychoanalytic community. Some argue that the Oedipal complex is a culturally specific concept that does not apply to all societies, and it reinforces gender stereotypes and heteronormativity [21]. Despite the ongoing debate, the Oedipal complex remains a central concept in psychoanalytic theory and continues to shift and influence the understanding of human psychology, desire, and development [21].

The tripartite theory of the psyche

The id represents our primitive impulses and desires, including sexual and aggressive urges. According to Freud, the id is the source of our unconscious thoughts and wishes and operates on the principle of immediate gratification [22]. For instance, consider a situation where one child observes another child enjoying their favorite snack. In response, the first child impulsively snatches the snack and swiftly consumes it. This impulsive act reflects the id’s instincts and desires. On the other hand, the ego serves as the rational and conscious aspect of the psyche, acting as a mediator between the id’s demands and the realities of the external world [22]. Its role is to satisfy the id’s needs in a socially acceptable manner, avoiding negative consequences. In our previous example, the child witnessing another child devouring their favorite snack would refrain from forcibly taking it, as doing so would result in punishment from an authority figure. Instead, the child might negotiate, offering a piece of their own snack in exchange for a portion of the other child’s snack, thus achieving their goal in a socially acceptable manner. Lastly, the superego symbolizes the internalization of societal norms and values [23]. It functions as a moral compass, determining right from wrong based on cultural and ethical standards. The superego enforces moral principles and may elicit feelings of guilt or shame when one’s actions violate those standards [22,23]. In the hypothetical scenario, the child may be tempted to steal the snack but recalls the moral teachings of their favorite cartoon character, which emphasizes that stealing is morally wrong. As a result, the child refrains from stealing the snack, influenced by the cultural impact of their beloved cartoon character [23].

Some argue that Freud’s tripartite theory oversimplifies human thought and behavior [24]. According to his theory, only three components underlie all the behavior a person will ever exhibit in their lifetime. His theory does not allow change or growth as an individual would continuously operate on at least one of these three principles [24]. According to Freud’s theory, humans are inherently selfish creatures who are always trying to manipulate a situation to attain their desires that will not bring punishment or pain [23-25]. Despite these criticisms, the id, ego, and superego concept contributes significantly to psychoanalysis [24]. It emphasizes the dynamic relationship between our primitive desires, rational consciousness, and societal norms. While it has been criticized for its narrow focus and deterministic view of the human psyche, it remains a significant contribution to psychoanalysis.

The secret world of dreams: Sigmund Freud’s interpretation and analysis

“The Interpretation of Dreams” (published in 1899) is considered one of the most influential books. In “The Interpretation of Dreams,” Freud provides insights into his theories about the mechanisms that underlie the unconscious mind [25]. He argued that the content of dreams is symbolic and that it is necessary to decode the symbolism to understand what the dream truly represents [25].

For Freud, dreams are a way for people to address their unconscious wishes. These wishes are unacceptable to our conscious mind and thus remain trapped in the recesses of the subconscious [26]. During sleep, the boundary between consciousness and unconsciousness becomes blurred, and therefore, we are allowed to address our repressed emotions within our dreams [26,27]. Freud believed that the manifest content of the dream (what we remember upon waking) was a disguise for the latent content (the true meaning of the dream) [26]. For example, in a dream where a person is flying, flight represents the desire to escape from a difficult situation or a yearning to experience freedom. A dream about a mouse might represent repressed feelings of inadequacy or weakness compared to others. Freud argued that dreams are a means of processing unresolved psychological conflicts. Thus, for Freud, dreams are a form of therapy that allows people to work through their difficult emotions and subconscious issues in the comfort of their own beds [26,27].

Unlike his other theories, Freud’s dream interpretation theory was the center of controversy. Some argued that his ideas were too focused on sexual desires and his approach was too subjective to the interpreter [27]. How can one tell if a dream has some deep underlying meaning or is just a dream? Others questioned the validity of interpreting dreams as a means of uncovering unconscious thoughts and emotions [27]. Like the tiger stalking the gazelle example discussed earlier, two therapists may interpret the same dream differently. There is no objective way to determine whose interpretation is correct. Despite these criticisms, Freud’s theory of dream interpretation remains a significant contribution to psychoanalysis. It has been used to gain insights into the workings of the unconscious mind and to treat various psychological disorders, including anxiety, depression, and post-traumatic stress disorder [27].

Going rogue with the mind: the psychoanalytical mavericks who challenged Freud

In the 1950s, psychoanalysis was not only established as its own discipline but was now being taken to new frontiers. Freud’s original ideas were challenged and built upon by a new generation of psychoanalytical theorists, including Carl Jung, Alfred Adler, Erik Erikson, and Karen Horney. These theorists emphasized different aspects of the human unconscious. The unconscious was used to address the mental health concerns of patients worldwide. In addition, therapy and healing became a central aspect of psychoanalysis.

Jung vs. Freud: even the deepest relationships cannot escape the idiosyncrasies of the ego

Carl Jung (1875-1961) was a Swiss psychiatrist, psychoanalyst, and former student of Freud who developed his own Analytical Psychology theory. While he was initially a follower of Sigmund Freud, his ideas eventually diverged from Freud’s, and the two men had a falling out [28]. Jung viewed classical psychoanalysis as a perspective that does not foster individual growth, nor did it provide the framework for the commonality of all people. Analytical Psychology is a psychological theory and framework that explores the collective unconscious, archetypes, and the process of Individuation [28,29]. Carl Jung recognized the existence of a shared reservoir of universal human experiences and symbols, known as the collective unconscious, which influences our thoughts, feelings, and behaviors through archetypes [30,31]. Individuation, a central concept in Jungian psychology, involves integrating both conscious and unconscious aspects of one’s personality to achieve wholeness and self-realization [31]. Like Freud, Jung saw dreams as a gateway to the unconscious mind [28]. Overall, Jungian psychology offers a holistic approach to understanding the human mind. Jung’s approach fosters personal growth and embraces a journey toward self-discovery. The following section will highlight the similarities and differences between Analytical Psychology and Freud’s Classical Psychoanalysis.

Similarities Between Jung’s Analytical Psychology and Psychoanalysis

The importance of the unconscious: Both Jung and Freud agreed that the unconscious mind plays a vital role in shaping our behaviors and experiences. They also believed that unconscious conflicts lead to psychological distress. Finally, they agreed that exploring the unconscious through free association could lead to spiritual and psychological healing [28]. While sharing these foundational beliefs with Sigmund Freud, Jung expanded upon them and introduced his own distinctive concepts. Jung argued that the unconscious had various aspects and was not solely driven by sexual motivations, as proposed by Freud. Rather the existence of a collective unconscious, a reservoir of shared human experiences, emotions, and symbols that shape our thoughts and behaviors through archetypes (we will discuss archetypes shortly), explained much of our behavior, if not all [29]. He saw the unconscious as a rich source of wisdom, creativity, and spiritual insight. Freud’s focus on repressed memories and painful truths about ourselves vastly differed from Jung’s, who explored the deepest layers of the psyche to integrate and harmonize conscious and unconscious aspects of one’s self to achieve personal growth and wholeness [29]. Jung’s views on the unconscious were not limited to pathology. Still, they encompassed a broader approach to understanding the human mind and its relation to universal themes, the cosmos, and archetypal patterns [29].

The use of dream analysis: Both Jung and Freud agreed that dreams are a window into the unconscious mind. They also decided that analyzing the symbols and themes within someone’s dreams could uncover repressed thoughts and feelings causing pain and strife in that person’s life [28]. Carl Jung was profoundly interested in dream analysis. His exploration of the unconscious through dream analysis was driven by his desire to bridge a gap between religion and science [29]. In his book “Memories, Dreams, Reflections,” Jung discusses how religion, science, and the unconscious are intertwined. He compares the term “the unconscious” with Gods, religions, and numinosity that have been present throughout human history [29]. Jung acknowledges that certain experiences, such as dreams and inspirations, arise spontaneously and cannot be attributed solely to conscious effort. He suggested that these experiences emanate from the human psyche, a demon, a god, or the unconscious [29]. Jung believed that the unconscious contains an unknown realm, parallel to reality as we know it, a vast expanse beneath the surface of consciousness. He acknowledged the limitations of scientific knowledge about the unconscious and the futility of objectively studying the workings of the unconscious. Like Freud, Jung’s approach to dream analysis sought to uncover the deeper meanings and symbolic messages embedded in dreams, considering them valuable sources of insight into the unconscious aspects of the individual’s psyche [29]. He embraced the subjective nature of dream analysis and accepted that although not perfect, it offers much to the therapist in addressing the psychological issues of their patients [29].

The emphasis on the therapist-patient relationship: Both Jung and Freud believed that the relationship between therapist and patient was key to the success of psychotherapy. They agreed that the therapist is a guide to help navigate unconscious conflicts and promote healing [28]. Carl Jung heavily emphasized the therapeutic alliance. He saw the relationship between the therapist and the patient as a crucial determinant for facilitating healing and growth. According to Jung, the therapist’s ability to establish a strong connection and rapport with the patient is paramount for a successful therapeutic process. He believed that a trusting and collaborative alliance allows the patient to feel safe, understood, and supported, creating a conducive environment for exploration and self-discovery [29]. His stress on creating a safe haven for patients to express themselves freely goes hand in hand with Freud’s free association.

Emphasis on the therapeutic alliance as a partnership is central to analytical therapy. The therapist must provide guidance, support, and overall compassion. Encouraging active engagement is crucial to success, according to Jung [28,29]. He underscored the value of empathy, listening actively, and caring. By demonstrating respect, acceptance, and a non-judgmental attitude, the therapist creates an environment that allows the patient to freely express their thoughts, real feelings, and true emotions [29]. The similarities between Freud’s emphasis on creating a safe environment and Jung’s emphasis on empathy highlight that Jung’s theories, although unique from Freud’s, remain rooted in classical psychoanalysis. Jung also believed that the therapeutic alliance provides the foundation for exploring the unconscious aspects of the patient’s psyche. He recognized that the unconscious holds valuable insights and symbols that can shed light on the patient’s inner world. Through collaboration and trust, the therapist can guide the patient into navigating and understanding the facets of their psyche. Ultimately uncovering hidden patterns, unresolved conflicts, and potentiating self-growth were the goals of Carl Jung [29].

Differences Between Jung’s Analytical Psychology and Psychoanalysis

The structure of the psyche: While both Jung and Freud emphasized the importance of the unconscious mind, they disagreed about its structure. Freud saw the psyche as consisting of the id, ego, and superego (as discussed earlier). Jung, in contrast, saw the psyche as consisting of three different layers: the conscious, the personal unconscious, and the collective unconscious. According to Jung, the conscious mind is just the tip of the iceberg and is limited in its ability to understand our true nature [29]. The conscious is what we are actively aware of and thinking about at the moment. The personal unconscious is the second layer of consciousness, which consists of all the experiences, memories, and feelings that are not within our immediate awareness. For example, a person may have an upsetting and recurring dream which initially appears to have no meaning. However, upon exploration in analytical therapy, the meaning behind this recurring dream may be discovered and addressed. This idea is very similar to Freud’s latent and manifest dreams. The personal unconscious also includes forgotten memories, repressed thoughts, and feelings we have not fully processed or are ready to acknowledge. Jung believed that the personal unconscious is a fundamental part of our psyche that could house the positive and negative components of the self [29]. The third and deepest layer of consciousness is the collective unconscious. Here we find the archetypes, symbols, and universal experiences that all humans share, regardless of dominion. Here, according to Jung, is the foundation of human thinking. It is the source of our deepest spiritual and creative impulses and what defines us as humans [29]. Jung believed that the collective unconscious was responsible for producing myths, fairy tales, and other universal symbols found in cultures and civilizations worldwide. Even day-to-day interactions can be explained with Jung’s theory. For example, a traffic light that uses green to symbolize *go* and red to symbolize *stop* may be defined by some deeply shared human positive emotion toward the color green and reservation to the color red. He believed that by tapping into this deeper layer of consciousness, individuals could better understand their psyche and the world around them [29].

What motivates people: Freud believed that repressed sexual desires universally lead to psychological distress and are the primary motivation source for human behavior [12,13]. Analytical psychologists argue that sexual desire is just one aspect of a broader human desire termed “Life Energy.” While acknowledging that sexual conflicts may be a source of distress, Jungian psychologists do not see sexual desire as the essential source of conflict for most people [28,29]. Instead, Life Energy is the primary psychic need that motivates us all to grow and pursue fulfilling lives [30]. Jung saw Life Energy as the fundamental aspect that drives individuals toward self-realization and wholeness [29]. Life Energy encompasses all forms of psychic energy, including creative needs, spiritual pursuits, and intelligence desires [28,29]. Jung saw the expression of life energy as being closely tied to a process he termed “Individuation,” which involves the integration of all aspects of self to reach a state of peace and tranquility [29]. He believed that Life Energy drives all humans toward pursuing Individuation. Life Energy manifests itself in various ways, such as creative pursuits, meaningful relationships, and spiritual practice. Jung saw the suppression of Life Energy as harmful to our well-being. People who suppress their Life Energy may experience physical or psychological symptoms [28,29]. Thus, unlike Freud, who viewed the unconscious as a place of shame and distress for his patients, Jung saw the unconscious as a gateway to reaching a state of Individuation or idealistic self.

Archetypes: According to Carl Jung, archetypes are recurring universal human experience themes rooted within the collective unconscious [29]. Archetypes represent fundamental aspects of human existence, such as characters, symbols, or situations, and they are shared across different cultures and periods [30,31]. For example, weddings have occurred in cultures worldwide despite no prior interaction between most cultures. It would seem then that getting married is a normal aspect of being human, which is shared among the collective unconscious of all people. Archetypes, therefore, arise from the innate psychological predispositions of humankind. They can manifest in arts, religion, literature, mythology, and poetry [32]. For example, the “Hero Archetype” has existed in numerous cultures throughout history. This is the story of a protagonist who must accomplish a quest to attain a goal, from Babylon’s Gilgamesh to today’s Hollywood movies. No culture has ever existed without a story that involves the “Hero Archetype.” Archetypes provide organizing patterns of thinking that shape our thoughts. Archetypes reflect shared human nature, providing a framework for interpreting the world [32]. By recognizing and working with archetypes, psychologists can gain insight into integrating shared unconscious elements into conscious awareness to provide effective therapy [30-32].

Individuation: Individuation is a central theme in analytical psychology. It refers to the psychological process of integrating all the aspects of oneself and thus realizing one’s true potential [31]. It requires putting together the conscious and unconscious elements of the mind. For Individuation to occur, one must strive to develop a unique identity and establish a harmony between conscious thoughts and unconscious desires and instincts [31,32]. It is a lifelong process that involves self-reflection, self-discovery, and self-acceptance, in that order [31]. Individuation goes beyond conforming to societal expectations. Jung highlights the need to explore inner depths and integrate the repressed aspects of the self no matter how shameful [31]. Through Individuation, individuals become more self-aware and authentic. The ultimate goal is to become in tune with oneself and one’s purpose. It is a journey of transformation that leads to personal fulfillment and a more meaningful life [30-33]. Although Sigmund Freud did acknowledge the importance of personal growth, he differed from Jung’s concept of Individuation. Freud’s goal was to alleviate psychological distress through the process of psychoanalysis. He never emphasized achieving a sense of wholeness or integration of the self [29,29,33].

While Carl Jung was initially a follower of Sigmund Freud, he eventually developed his theory of analytical psychology and quickly diverged from Freud’s classical psychoanalysis. While there are a few similarities between the two, such as the emphasis on the unconscious and the use of dream analysis, there were also poignant differences, such as their views on what drives humans, the structure of the psyche, the concept of self, and the ultimate goal of humans. Freud emphasized the significance of sexual desire for driving human nature, but Carl Jung disagreed. He believed sexual needs were a small part of a vast human desire called Life Energy. Life Energy is the desire to attain Individuation, a state of self-realization. Individuation occurs when the collective unconscious, personal unconscious, and conscious work harmoniously to acknowledge all aspects of oneself.

The battle of Vienna: Adler vs. Freud - feelings of inferiority

Alfred Adler (1870-1937) was an Austrian psychiatrist, psychotherapist, and former student of Sigmund Freud. Like Jung, he developed his theory rooted in psychoanalysis, known as Individual Psychology. Adler began as a follower of Sigmund Freud. Similarly to Jung, he eventually developed his approach to psychoanalysis. Individual Psychology emphasizes the holistic nature of people and considers the interplay between the psychological, biological, and cultural factors for shaping feelings, thoughts, and cognition. Adler emphasizes the importance of understanding an individual’s perspective to explain why they are the way they are [34,35]. The Adlerian theory emphasizes personal growth and self-improvement, similar to Jungian psychology. Individual psychology, however, highlights the concept of inferiority and superiority in an individual’s development [34]. Adler heavily emphasized negative feelings from childhood stemming into the strife individuals experience in adulthood. Individual psychology offers a unique framework for understanding individuals as striving beings influenced by their social environments [34,35]. In this response, we will summarize Adler’s research concerning psychoanalysis, highlighting both the similarities and differences between the two approaches.

Similarities Between Adler’s Individual Psychology and Psychoanalysis

The importance of childhood experiences: Both Adler and Freud agreed that early childhood experiences shape personality, self-image, and behavior in adulthood. They agreed that the therapist should help the patient explore and understand early life experiences to gain insight into their current strife [34]. Adler, just like Freud, saw childhood as pivotal. He recognized that early interactions with caregivers and the social environment impact a person’s sense of self-worth and ability to navigate life’s obstacles [34]. Adler, unlike Freud, highlighted the importance of empowering children to develop a sense of competence, confidence, and mastery of tasks. He encouraged children to overcome feelings of inferiority and contribute positively to society. Adler’s holistic approach to childhood and development emphasized the interplay between social dynamics, individual aspirations, and the cultivation of social interest as crucial factors in promoting healthy psychological growth [34].

The use of free association: Both Adler and Freud used free association to access the unconscious thoughts of their patients. They both agreed that exploring the unconscious aspects of the mind could lead to greater self-awareness and, ultimately, healing [34]. Through Adler’s observations of children affected by “organic deficiencies” (physical handicaps), he established his theories on Superiority and Inferiority. He found that children who experienced physical limitations needed to compensate and achieve a feeling of superiority over other children [35]. This striving for superiority propelled them to take on more significant challenges in life and perceive the world as enemy territory [35]. For example, someone with no arms walking behind you may be offended that you opened the door for them despite being willing to open it for anyone. Perceiving that you opened that door for them because of their organic deficiency, they would be more inclined to take on the challenge of opening the door themselves than someone with arms to prove they can. Many of the actions these children performed became part of their adaptive response to their deficiency [35]. According to Adler, these children quickly and firmly established defensive and offensive attitudes, developing antagonistic behaviors such as fighting, hesitating, stopping, and pushing [35]. Adler argued that through intense focus on themselves and their flaws, they tend to be egocentric, lacking social empathy, courage, and self-confidence, as they fear defeat more than they desire success [35]. These children actively sought out favorable situations while creating barriers to avoid confronting challenges that they may not be able to surpass [35]. These children, burdened by the inferiority of their organs, became strongly influenced by the darker aspects of life [35]. In his therapeutic work, Adler utilized free association to explore and unravel these unconscious dynamics, helping these children gain insight into their thought patterns and strategies for compensating for their deficiencies, ultimately supporting them in developing a healthier and more socially connected life [34-36].

The therapeutic relationship: Adler, Jung, and Freud agreed that therapeutic alliance is critical to success and change in at-risk children. They also all agreed that the therapist must establish trust and nurture a relationship based on openness and willingness to communicate. He underscored facilitating a safe space for exploration and growth [34,35]. According to Adler, the importance of a therapeutic alliance extends beyond the boundaries of the therapist and patient. Adler believed that therapists, social workers, and teachers all play roles in addressing the psychological issues of children and thus must work together as a team [35]. Therapists can guide children toward a more hopeful future by establishing a strong partnership with parents and collaborating with teachers. This future fosters the growth of individuals who have become accountable, aware, and willing to give back to society [35]. Adler’s emphasis on the therapeutic alliance highlights the need for a community effort to shape the development of children [35].

Differences Between Adler’s Individual Psychology and Psychoanalysis

The drive of human nature: Freud saw sexual desire as the primary motivation of human behavior. Jung saw Life Energy as the ultimate drive, but Adler believed that striving for superiority was humankind’s primary motivation. Individual psychology sees people as inherently needing to overcome feelings of inferiority. Just like Jung, Adler acknowledged that sexual problems could be a source of psychological distress at times. He did not emphasize them [35,36]. He believed that people are motivated by a need to overcome their feelings of inferiority and thus strive for superiority. He termed this ambition the “will to power.” According to Adler, everyone experiences a sense of inferiority stemming from childhood experiences of inadequacy compared to others [36]. For example, a child who failed a math test would experience feelings of inferiority and would work extra hard to do well on the next math test to achieve a feeling of superiority. If the child continues to do poorly in math, they will carry on through life, avoiding math problems, thus preventing that feeling of being inferior. Therefore, feeling of inferiority provides all people with two choices, strive to become superior or avoid that obstacle that once made them feel inferior [36].

The concept of the unconscious: While both Adler and Freud believed in the importance of the unconscious mind, they disagreed about its structure. Freud saw the unconscious as a repository for repressed thoughts and feelings that were too painful to be brought into consciousness. Adler believed the unconscious is a source of creativity and problem-solving, unlike Jung [37]. Adler did not see the need to distinguish between the conscious and unconscious realms clearly [37]. He recognized the fluidity between levels of awareness, whereby what may initially seem unconscious can be raised to consciousness through effective therapy or when it becomes relevant and necessary. Many things remain unconscious because they are not immediately appropriate or needed in conscious awareness. However, these unconscious elements can be brought into consciousness when required. Adler understood that individuals tend to focus on and consider only those aspects supporting their self-enhancement goals. Elements disturbing or challenging their viewpoint are often left aside in the unconscious [37]. For Adler, the conscious mind becomes a source of encouragement, while the unconscious holds what might disrupt or hinder the individual’s perspective. The individual’s lifestyle, to some extent, reflects the degree of narrow or broad focus in their awareness. A narrow focus may disregard or suppress aspects not aligning with their goals.

In contrast, a more general perspective allows for a more comprehensive understanding and integration of conscious and unconscious elements [35]. Adler’s views on the unconscious align with his holistic assessment of the individual. He believed the mind could not be divided into separate and antagonistic halves of the conscious and unconscious. Instead, consciousness and unconsciousness are directed by the individual’s fictional final goal, representing their ultimate purpose and self-enhancement [38]. Self-enhancement in this accord is not unlike his contemporary Carl Jung’s “Individuation,” both goals being continued self-improvement [31,32].

While there are similarities between self-enhancement and Individuation regarding personal growth, there are also nuances. Self-enhancement focuses on overcoming feelings of inferiority [37], emphasizing building a sense of superiority [37]. In contrast, Individuation is all about self-discovery and the journey to self-realization. Wholeness is established through incorporating all aspects of oneself [31,32]. Adler and Jung recognized the importance of self-actualization and fulfilling one’s potential. Ultimately, self-enhancement and Individuation represent two different approaches to growth. Self-enhancement focused on personal achievement and superiority, and Individuation focused on self-discovery, integration, and wholeness.

The concept of the self: Adler did not use the term “self” as Jung did. However, he agreed that people have a fundamental sense of self shaped by interactions with others and experiences. Thus, Adler saw the goal of therapy as helping others develop a more positive sense of self and enabling them to overcome feelings of inferiority [34-36]. Adler’s concept of self emphasizes the indivisibility of the mind rejecting a notion of a boundary between the conscious and unconscious. Instead, both are guided by the individual’s final goal, which drives their decision-making [37]. According to Adler, the opposition between conscious and unconscious impulses is merely a difference in means, with both aspects ultimately working toward enhancing the self. He argued that there is fluidity between levels of awareness. Thus, at one time, the unconscious may be raised to consciousness when necessary [37]. Adler recognizes that not everything must be consciously attended to at all times. Certain thoughts and feelings may remain unconscious until they become relevant or required to undergo a course of action [37]. The conscious mind focuses on thoughts, feelings, and experiences that support and reinforce the patient. The unconscious mind possesses ideas and feelings that disrupt or challenge their perspective of themselves [37]. Overall, Adler’s self-concept highlights the interconnectedness of conscious and unconscious processes. The fluidity between these levels of awareness suggests that unconscious elements can be made conscious, and a comprehensive understanding of the self can be achieved through effective therapy and self-reflection.

Alfred Adler and Sigmund Freud were two of the most influential figures in the development of modern psychology, yet their theories differed significantly. While Freud focused on the unconscious mind and the role of instinctual drives, Adler saw the importance of needing to feel superior. While working with children with organic deficiency, Adler noticed that these children strived to put themselves in situations where they could feel superior and avoid problems that made them feel inferior [35]. His observations led Adler to believe that all people have feelings of inferiority that stem from childhood. Adler also emphasized the self more than Freud did. Adler believed that by combining the positive ideas of one’s self from the conscious mind and negative aspects from the unconscious mind, one could reach enlightenment and self-awareness.

Emphasizing stages: Erikson and Freud’s views on personality development

Erik Erikson (1902-1994) was a psychologist who built on Freud’s theories and developed his approach to psychosocial development. Erikson states that we all must overcome eight stages of development. These stages encompass a range of challenges, such as establishing trust with caregivers, overwhelming feelings of guilt, forming an identity, and accomplishing autonomy. The Eriksonian theory emphasizes achieving a healthy balance between societal demands and our own needs. Thus, he focuses on the societal and cultural factors that influence us at every point of our lives [38]. Each stage is characterized by a specific crisis that individuals must resolve to mature and become successful and content with life [39,40]. By addressing the psychosocial challenges of each stage, individuals have an opportunity to establish a fulfilling life. We will summarize Erikson’s theory, highlighting the similarities and differences between him and Freud [38].

Similarities Between Erikson’s Psychosocial Theory and Psychoanalysis

The importance of early childhood experiences: Like Freud, Erikson agreed that early childhood experiences have a lasting impact on personality development. Like Freud, he emphasized the importance of the first few years of life in shaping a person’s sense of self [38-40]. Classical psychoanalytic theory heavily emphasizes childhood experience, particularly the psychosexual stages [14]. Freud argued that the experiences of the first few years of life, especially interactions with parents or caregivers, ultimately shape an individual’s personality [14]. Erikson expanded on Freud’s emphasis on psychosexual development to encompass a lifelong journey with stages marked by developmental tasks. Erikson did agree with Freud in regards to childhood experiences being pivotal. However, he extended the focus to include the entire lifespan [38]. They both agreed and recognized that early childhood experiences impact an individual’s psychological development and personality. They believed unresolved conflicts or challenges during these formative years could lead to long-term consequences [38,39]. Freud focused on the sexual drives that occurred during infancy and early childhood, but Erikson believed every stage of life has its own drive, and these drives extend passed just early childhood [14].

The influence of the unconscious mind: While Erikson acknowledged the existence of the unconscious and believed it does play some role in processing information, he did not focus on it or speak about it much [40]. Unlike Freud, Jung, and Adler, Erikson was indifferent to the unconscious processes. He did not acknowledge them much in his developmental theory. For Erikson, the conscious mind plays a prominent role, and unconscious processes are irrelevant at best [40]. Erikson believed that individuals actively engage in self-discovery and identity formation, which involves conscious reflection [40]. While Erikson did not dismiss the existence of unconscious influence, he placed heavier emphasis on conscious awareness and how this conscious awareness allowed for integrating social and cultural factors [40].

Differences Between Erikson’s Psychosocial Theory and Psychoanalysis

The drive of human nature: According to Freud, humans are driven by sexual urges, particularly in childhood. Erikson disagreed. For Erikson, every part of life presents its own unique challenge that causes people to purpose [40]. Erikson’s theory focused more on social and emotional development than the sexual aspects emphasized by Freud [41]. However, a stage in Erikson’s view is often associated with Freud’s emphasis on sexuality, and that is Erikson’s “Identity vs. Role Confusion” stage, which occurs during adolescence (12 to 18 years old). During Erikson’s Identity vs. Role Confusion stage, adolescents explore their identities, trying to establish a sense of self and figure out their place in society [42]. This stage is characterized by the search for a cohesive identity, including exploring one’s sexual and gender identity. Thus, while Erikson did not emphasize sexual development as Freud did, this stage does involve exploring sexual and romantic relationships, which, in a way, can be seen as parallel to Freud’s focus on sexual development [42].

The focus on social and cultural influences: Erikson emphasized the role of social and cultural factors in shaping personality development. Freud rarely acknowledged or spoke about the role of society or culture in his theoretical approach. Erikson believed that a person’s social context played a significant role in shaping identity [40]. The fourth stage, “Industry vs. Inferiority,” exemplifies the cultural focus in development. This stage occurs between the ages of six and 12 [42]. In the Industry vs. Inferiority stage, children develop a sense of competence in various social and academic settings. The child must strive to acquire new skills, accomplish tasks, and receive recognition and praise from others. A parallel between Adler’s Will to Power can be seen in this stage, as the child must strive to feel superior at this point in their life.

Interestingly, many of the children that Adler worked with were between the ages of six and 12 when he initially formulated his theory [35]. According to Erkison [42], this stage is crucial for developing self-esteem. Culture significantly influences the activities and expectations during this stage. Educational systems, societal norms, and cultural practices shape the tasks and activities children are encouraged to pursue. Cultural factors also influence the standards against which children measure their competence. Cultural values, beliefs, and social comparisons shape children’s perceptions of success or failure. By acknowledging the role of culture in the Industry vs. Inferiority stage, Erikson’s theory recognizes that cultural contexts influence children’s socialization and self-evaluation [42].

The emphasis on stages of development: Erikson’s theory proposes eight stages of psychosocial development, each characterized by a particular crisis or challenge that must be resolved for them to progress to the next stage [42]. These stages are influenced by social factors such as family, peers, cultural norms, and the individual’s personality and temperament. For example, in the first stage of psychosocial development, trust vs. mistrust, infants must learn to trust their caregivers to develop a sense of security and basic trust in the world around them [42]. If caregivers are consistently responsive and meet the infant’s needs, the child will develop a sense of trust, influencing their future relationships and sense of self. However, if caregivers are unresponsive or inconsistent, the child may develop a sense of mistrust, which could lead to future difficulties in forming trusting relationships.

Similarly, in the fifth stage of psychosocial development, identity vs. role confusion, adolescents must navigate the challenges of identity formation and establish a sense of self-consistent with their cultural and social context [42]. If adolescents can successfully integrate their personal values and goals with the expectations of their social environment, they will develop a sense of identity and purpose. However, they may experience confusion and a lack of direction if they cannot do so. According to Erikson, each of the eight stages of development has its own unique set of challenges [42].

Erikson’s theory of psychosocial development proposed eight stages of development, each characterized by a particular crisis or challenge that must be resolved to progress to the next stage. Unlike Freud, Erikson believed that development continues throughout the lifespan and that successful resolution of these stages leads to a more integrated sense of self. Additionally, Erikson’s theory emphasized the potential for personal growth and change throughout the lifespan rather than the fixed nature of personality proposed by Freud. While Freud stresses the role of the unconscious mind and the importance of sexual and aggressive drives in shaping personality, Erikson believed that social and cultural factors played a more significant role.

Beyond the Oedipus complex: Horney’s feminist challenge to Freudian psychoanalysis

Karen Horney (1885-1952) was a psychoanalyst who developed her own theories about the unconscious influences and mechanisms that shape and ultimately determine behavior [43]. Horneyian psychology offers an alternative perspective from traditional psychoanalysis. Like Erikson and Jung, she emphasized the influence of cultural elements that lead to internal conflict [43]. Horney introduced the “interpersonal theory.” She highlights the importance of early relationships in shaping someone's personality [44]. In this regard, social and cultural context is crucial in understanding human behavior and the emergence of what she termed “neurotic patterns.” At the core of Horney’s theory is “basic anxiety.” Individuals develop a fundamental sense of insecurity and anxiety during their early years due to feelings of helplessness and vulnerability, similar to Adler’s inferiority complex [44,45]. According to Horney, we are all imprinted with certain gender roles, which leads to feelings of internal strife. This anxiety influences the individual’s subsequent psychological development and contributes to the formation of neurotic behaviors to cope with the stressors of day-to-day living.

Similarities Between Karen Horney’s Theory and Psychoanalysis

The importance of early childhood experiences: Like all the other psychoanalysts we discussed, Horney agreed that early childhood experiences play a significant role in shaping a person [43]. Unlike Freud, who emphasized the influence of unconscious drives, conflicts, and sexual development [1-3], Horney focused on the impact of gender roles and sexuality in forming an individual’s sense of self and their relationship to society [43,44].

Karen Horney and Sigmund Freud share similarities in their views on the unconscious [1-3,43,44]. Both psychologists acknowledged the existence of the unconscious mind, recognizing that individuals are not always aware of the motivations, desires, and conflicts that drive their thoughts and behaviors. They agreed that the unconscious significantly influences human behavior, shaping aspects of personality, emotional experiences, and the formation of psychological symptoms [43,44]. While their theories diverged in specific mechanisms and influences on the unconscious, such as cultural and social factors in Horney’s approach versus innate drives in Freud’s theory, they recognized the importance of exploring and understanding the unconscious to gain insight into human psychology [43,44].

Differences Between Karen Horney’s Theory and Psychoanalysis

The role of culture and society: Horney believed that cultural and societal factors played a significant role in shaping personality, whereas Freud’s psychoanalytic theory focused primarily on the individual psyche. She emphasized the importance of social influences such as gender roles, cultural norms, and societal expectations in shaping a person's sense of self [43,44]. Horney believed that women, in particular, are socialized to adopt submissive and nurturing roles, which can lead to feelings of inferiority and helplessness down the line [43]. Women are often taught to prioritize the needs of others over their own, which can lead to a lack of self-esteem and a sense of being powerless or dependent on others. Horney believed that this cultural conditioning could lead to the development of what she called “feminine psychology,” which is characterized by a preoccupation with love, relationships, and the desire for approval from others [44]. Women who internalize these cultural messages may struggle with insecurity and self-doubt, affecting their sense of self and ability to achieve their goals [43,44]. Horney also believed that men are subject to cultural expectations and that traditional male gender roles can lead to emotional detachment and a need to prove oneself through competition and achievement. Men may struggle with feelings of isolation and a sense of disconnection from their emotions, which can affect their ability to form intimate relationships and achieve a sense of fulfillment in their lives [43,44]. Karen Horney and Alfred Adler had contrasting views on the concept of inferiority while sharing some similarities. Horney believed that feelings of inferiority stem from societal pressures and parental attitudes, leading to inadequacy and insecurity [44,45]. She emphasized the role of external validation and success as compensatory strategies to overcome these feelings.

In contrast, Adler viewed inferiority as an inherent part of the human condition, driving individuals to strive for superiority and personal growth. He emphasized the importance of overcoming inferiority through developing a sense of community and contributing to society [35,36]. Both theorists recognized the impact of childhood experiences on the development of inferiority. Still, Horney focused more on cultural and social influences, while Adler emphasized individual psychology and the pursuit of social interest.

The role of anxiety: Horney placed a greater emphasis on the role of anxiety in shaping behavior and personality than Freud did. She believed anxiety was a normal and necessary part of life and could be harnessed to promote growth and change [45]. Horney developed the concept of “basic anxiety,” which refers to a deep-seated feeling of insecurity and helplessness that arises from early childhood experiences. Basic anxiety can lead to neurosis and other psychological problems if not addressed. Horney believed that basic anxiety was a pervasive sense of vulnerability and insecurity from early childhood experiences, such as neglect, rejection, or criticism from caregivers [45]. This basic anxiety can become a central aspect of an individual’s personality and affect their relationships, sense of self, and ability to cope with stress. According to Horney, there are several ways in which individuals may respond to basic anxiety. One response is to seek security and protection from others, which can lead to a preoccupation with relationships and a fear of rejection or abandonment. Another response is to develop a strong sense of independence and self-sufficiency, which can lead to a fear of being controlled or dominated by others. A third response is to become aggressive or domineering, which can be a defense against feelings of helplessness and vulnerability [45].

The concept of neurotic needs: Horney identified the “neurotic needs” common to everyone and could lead to neurosis if not addressed. These included the need for affection and approval, the need for power and control, and the need for perfection as well as others [45]. According to Horney, neurotic needs are compulsive desires or behaviors that individuals adopt to feel more secure and alleviate anxiety. These needs are not based on genuine personal interests or values but rather on a perceived need to conform to the expectations of others or to achieve a sense of control over their environment [45]. These needs can become so ingrained in an individual’s personality that they interfere with their ability to form healthy relationships, pursue personal interests, and achieve a sense of fulfillment in life. Horney believed that the development of neurotic needs was influenced by early childhood experiences, particularly those that involved feelings of neglect, rejection, or criticism from caregivers. These experiences can make individuals feel insecure and vulnerable and adopt behaviors or attitudes that they believe will protect them from further harm [45].

The role of psychotherapy: Horney’s approach to psychotherapy differed significantly from traditional psychoanalysis. She believed the therapist’s role was to create a supportive and empathic environment where the client could explore and understand their emotions and experiences rather than interpret and analyze the client’s unconscious motivations and conflicts [43,44].

Karen Horney and Sigmund Freud had significant differences in their personality and psychological development theories. Freud emphasized the importance of innate drives and instincts in shaping an individual’s psychological development. At the same time, Horney believed that an individual’s personality was largely shaped by their social and cultural experiences, particularly their early relationships with caregivers. Horney also differed from Freud’s views on gender and sexuality, emphasizing that gender roles were socially constructed and influenced by cultural norms and expectations. Overall, while Freud’s theories continue to be influential in modern psychology, Horney’s emphasis on the role of social and cultural experiences in shaping an individual’s personality has had a lasting impact on psychology.

Strengths, limitations, and contributions of this study

One of the major strengths of this review paper is the comprehensive coverage of the major contributors to the field of psychoanalysis and how their views contrast with the founder, Sigmund Freud. By exploring the findings of these contributors, the reader is offered many unique perspectives and, thus, a broader understanding of the field. However, a limitation of this review is its lack of detailed exploration of the theories and concepts put forth by these major contributors. Due to the nature of the paper, which aims to provide an overview of the history of psychoanalysis, it does not have the space to delve deeply into each theorist’s work. Consequently, readers seeking an in-depth analysis of specific theories may use this review as a starting place for their research and learning. We hope that this paper could put forth a framework for what psychoanalysis is and where it came from. In addition, we hope to have sparked the reader’s interest to further read and learn more about this fascinating psychology paradigm.

## Conclusions

The theories of Sigmund Freud have laid a foundation for psychoanalytical psychology. Although the thoughts and attitudes of his students differed from him, they remained grounded in psychoanalysis. All four psychoanalysts we have discussed agree that childhood plays a significant role in development. Freud, as we discussed earlier, emphasized the sexual nature of people and believed this was the most significant factor in determining a person’s behavior and personality. Jung spoke about the collective unconscious and the need for self-realization, a drive he termed “individuation.” In his theory, we all share a collective unconscious and must use the archetypes of this collective unconscious with our consciousness to attain self-realization. Adler had his version of self-realization, called “Will to Power.” Adler saw all people needing to overcome feelings of inferiority from childhood to attain feelings of superiority in adulthood. Erikson believed every life stage has unique challenges and drives to overcome negative feelings. Horney emphasized the feelings of anxiety influencing our behavior and drives.

While Freud spoke of the id, ego, and superego in his theory of the unconscious, this was not a central theme for his students. Jung highlighted the collective unconscious, a universal shared architecture of understanding the world around us. Adler saw the unconscious and consciousness as more fluid than Freud did. He believed people could more easily access the unconscious than Freud had originally postulated. Erikson acknowledged the unconscious but was indifferent about it in his theoretical framework. Horney’s view of the unconscious was very similar to Freud’s; however, she heavily emphasized how gender roles influence our unconsciousness. In conclusion, the diverse perspectives of Freud and his students, such as Jung, Adler, Erikson, and Horney, have contributed to the rich tapestry of psychoanalytical psychology, each offering unique insights into the role of childhood, the unconscious mind, and the factors shaping human behavior and personality.
